# The Effect of a Mindfulness-Based Intervention on Nurses in Kelantan, Malaysia

**DOI:** 10.21315/mjms2021.28.6.12

**Published:** 2021-12-22

**Authors:** Nor Asyikin FADZIL, Wei Ooi HEONG, Yee Cheng KUEH, Cheng Kar PHANG

**Affiliations:** 1 Department of Psychiatry, School of Medical Sciences, Universiti Sains Malaysia, Kubang Kerian, Kelantan, Malaysia; 2 Biostatistics and Research Methodology Unit, School of Medical Sciences, Universiti Sains Malaysia, Kubang Kerian, Kelantan, Malaysia; 3 Behavioural Health Centre, Sunway Medical Centre, Bandar Sunway, Selangor, Malaysia

**Keywords:** mindfulness, nurses, stress, anxiety, depression

## Abstract

**Background:**

Among healthcare workers, nurses have a particularly high risk of stress. Stressful conditions in the workplace increase the possibility of burnout and psychological distress. Short mindfulness-based interventions can help to decrease stress, anxiety and depression. This study aimed to explore the effectiveness of a mindfulness-based intervention in reducing perceived stress, anxiety and depression among public teaching hospital staff nurses.

**Methods:**

Thirty-five nurses from various specialities were recruited from Hospital Universiti Sains Malaysia (HUSM). The intervention comprised a 1-day brief mindfulness-based intervention workshop and 1 h group practice session each month for 3 months together with daily follow-up via WhatsApp group. All the participants completed a self-administered sociodemographic questionnaire validated for use in a Malay population. The Depression, Anxiety and Stress Scale 21 (DASS 21) and Perceived Stress Scale 10 (PSS 10) were used to measure perceived stress, anxiety and depression before the intervention, and 3 months later upon completion of the intervention.

**Results:**

There was a statistically significant reduction in the scores for stress perception (95% confidence interval [CI]: 0.06, 2.92;* P* = 0.04) and anxiety (95% CI: 0.06, 2.34;* P* = 0.04) post-intervention.

**Conclusion:**

A brief mindfulness-based intervention was effective in reducing perceived stress and anxiety among nurses.

## Introduction

Occupational stress is characterised by harmful physical and emotional responses when job requirements do not conform to workers’ abilities, resources or needs (1, 2). As the nursing profession faces high demands in terms of service provision, it is considered a particularly stressful job (1, 3). According to Ghawadra et al. (4), 41% of hospital staff nurses experience psychological distress. Zainiyah et al. (5) found similar findings, reporting that 24.6% of ward nurses in a Malaysian public hospital experienced stress. Thus, interventions for stress reduction are crucial for nurses.

Increasingly, research has focused on the effectiveness of mindfulness training in stress reduction (3–5). The concept of mindfulness emphasises present-moment awareness, in which a person purposefully pays attention to the present-moment experience in a non-judgemental way (6). Many people have a tendency to dwell on problems, both past, present and future. Based on past experience, they may catastrophise current life events, giving rise to stress and negative emotions (7). Paying less attention to the present may have adverse implications for work-related tasks and work output.

Kabat-Zinn (6) developed an 8-week mindfulness-based stress reduction programme. This programme involves mindful breathing exercise, training aimed at bringing attention to the present moment, listening attentively and cultivating emotional awareness in a non-judgmental way (8). It meets the specific needs of healthcare professionals by helping them to deal with stressful life events, improving present-moment awareness, decreasing distraction and helping them to control emotion in a stressful environment (9).

Previous studies reported that mindfulness-based interventions significantly improved mindfulness levels and reduced stress levels in the general population (2), as well as among specific populations, such as undergraduate students, medical students (10, 11) and healthcare providers (2, 8, 12–15) thereby enhancing well-being. A 5-week mindfulness-based intervention study among nurses at a critical care unit in Malaysia teaching hospital reported significantly reduced stress and improved levels of mindfulness and happiness post-intervention (15).

Although numerous studies have shown that mindfulness-based interventions help nurses to manage work-related stress (3, 13–19), a major obstacle to implementing mindfulness-based intervention training for the nursing profession is the time required for training and attending weekly practice sessions (8, 15, 19). Brief mindfulness-based interventions may help to overcome this obstacle. Gilmartin et al. (8) found that a brief mindfulness-based intervention based on the programme developed by Kabat-Zinn (6) improved perceptions of stress and well-being among healthcare workers. One-day brief mindfulness-based interventions require less time and cost to deliver than interventions of longer durations. This study aimed to assess the effectiveness of a 1-day brief mindfulness-based intervention based on a 5-week mindfulness-based intervention programme (MINDFULGym) developed previously (19) in reducing stress among nurses.

## Methods

### Study Setting and Subjects

This intervention study was conducted at Hospital Universiti Sains Malaysia (HUSM), a public teaching hospital in Kelantan, Malaysia for 3 months from 1 August 2018 to 31 October 2018. Nurses were invited to participate in the programme via flyers distributed throughout the hospital. Participants who volunteered to take part in the programme and fulfilled the inclusion criteria were recruited through purposive sampling. The inclusion criteria were at least certificate or diploma qualification in nursing, practising at HUSM, at least 3 years of working experience and involved in patient care duties. The exclusion criteria were working in outpatient clinics, participation in mindfulness training in the past, physical disabilities or severe mental illness.

The sample size was estimated based on a reference study (15) using power and sample size programme software, with α = 0.05, power = 80%, standard deviation of stress score = 5.75 and expected detectable difference in stress score = 3.0 from pre- to post-intervention. The total number of participants required was 35, assuming a 10% dropout rate.

### Description of the Intervention

**Figure 1:**
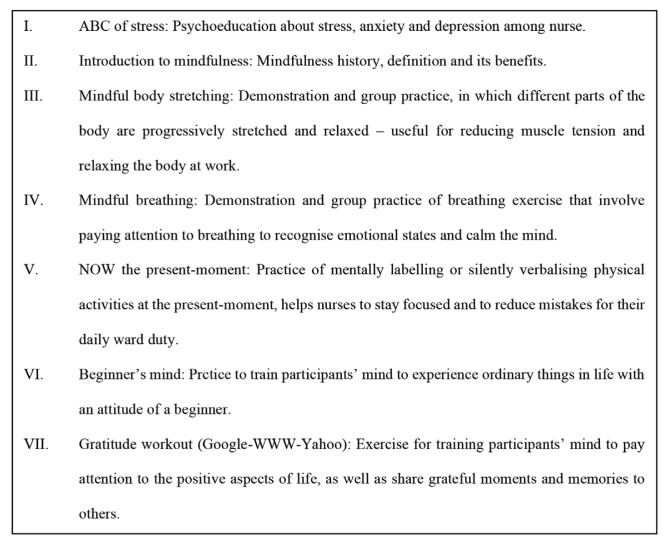
Outline of intervention workshop for mindfulness training

The 1-day brief mindfulness-based intervention was a short version of the MINDFULGym mindfulness-based intervention (19). All the participants took part in a 1-day mindfulness-based intervention workshop conducted by Dr Phang Cheng Kar (MINDFULGym creator) and ourselves. After the 1-day workshop, the participants took part in supervised 1 h practice sessions each month for 3 months. All the participants had to attend at least two group practice sessions. Each participant was given a copy of a mindfulness-based intervention modules handout to practise mindfulness-based intervention at home. An outline of the programme is shown in Figure 1. The participants were advised to practice and apply the mindfulness-based intervention exercises at work and home regularly. Adherence to the intervention was enhanced by daily group message reminders sent via WhatsApp. The participants were also encouraged to share their experiences, including photographs, during self-mindfulness practice or ask questions via the WhatsApp group.

### Measures

All the participants completed a self-reported sociodemographic questionnaire, as well as the Perceived Stress Scale 10 (PSS 10) and Depression Anxiety Stress Scale 21 (DASS 21). The PSS 10 and DASS 21 questionnaires were completed before the mindfulness-based intervention and again 3 months later at the end of the intervention.

### PSS 10

The PSS 10 was used to measure the impact of the intervention on stress. The PSS 10 is a 10-item questionnaire, which measures self-reported levels of stress during the previous month (20). Each item comprises a 5-point scale from 0 (never) to 4 (very often). Higher scores on this scale indicate higher levels of stress. The Malay-translated version of the PSS 10 was validated, with a Cronbach’s alpha value of 0.63 and intra-class correlation coefficient value of 0.81 (21).

### DASS 21

The DASS 21 is a 21-item questionnaire, which measures stress, anxiety and depression severity over the past week. Each of the three DASS 21 sub-scales contains seven items, which are divided into subscales with relevant items. The scores are multiplied by 2 to obtain the final total score. Based on the sub-scale scores, the item (stress, anxiety or depression) is classified as normal, mild, moderate, severe or extremely severe. For the purpose of this study, only the depression and anxiety scale were used. On the anxiety sub-scale, scores of 0–3 = normal, 4–5 = mild, 6–7 = moderate, 8–9 = severe and 10 and above = extremely severe. On the depression subscale, scores of 0–4, 5–6, 7–10, 11–13, and 14 and above denote normal, mild, moderate, severe and extremely severe, respectively (22). The Malay version of the DASS 21 was used in this study. It was translated into Malay and validated by Ramli et al. (23), with good Cronbach’s alpha values of 0.84, 0.74 and 0.79 for depression, anxiety and stress, respectively.

### Data Analysis

The data were analysed using the Statistical Package for the Social Sciences (SPSS) version 24.0. An intention-to-treat (ITT) analysis was conducted. Data were checked for a normal distribution. For data with that complied with a normal distribution, the mean and standard deviation (SD) are reported. Conversely, for data with a skewed distribution, the median and interquartile range (IQR) are reported. Differences in the means of the scores of the variables pre- versus post-intervention were analysed using a paired-samples *t*-test. The effect size was calculated using Cohen’s *d* formula. If the data were skewed, the non-parametric Wilcoxon signed-ranks test was used.

## Results

In total, 30 of the 35 participants completed the study. Five nurses did not complete the minimum requirement to attend two of the three monthly group practice sessions. The scores of these nurses were included in the final analysis as an ITT analysis in the study.

### Sociodemographic Data

The sociodemographic data and characteristics of the participants are summarised in Table 1. The mean age of the total sample was 34.63 years old. The mean number of years practising nursing was 11.09 (SD = 6.52). The majority of the nurses were Malay (*n* = 34, 97.1%), female (*n* = 33, 94.3%) and married (*n* = 31, 88.6%), and the majority held a diploma in nursing (*n* = 27, 77.1%) (Table 1).

### Pre- and Post-Intervention Changes in Perceived Stress Scores

Before the intervention, the perceived stress score mean for the total sample was 15.66 (SD = 5.75). Post-intervention, there was a marked improvement in the mean perceived stress score (14.17, SD = 4.32). Thus, stress perception was significantly reduced post-intervention (95% CI: 0.06, 2.92; *P* = 0.04) (Table 2).

### Pre- and Post-Intervention Changes in Anxiety Scores

A higher anxiety score was reported pre- (mean = 7.37*,* SD = 6.07) versus post-intervention (mean = 6.17, SD = 4.40), a reduction of 1.20 points. There was a significant decrease in anxiety post-intervention (*P* = 0.04), with a small effect size (Cohen’s *d* = 0.36) (Table 2).

### Pre- and Post-Intervention Changes in Depression Scores

There was no difference between the participants’ depression scores pre- (median = 4.00, interquartile range [IQR] = 6) versus post-intervention (median = 4, IQR = 4) based on Wilcoxon’s signed-ranks test (*P* = 0.62) (Table 2).

## Discussion

This study evaluated the effectiveness of a brief mindfulness intervention in reducing stress among staff nurses (*N* = 35) in HUSM, of whom 23% reported stress prior to the intervention. This level of stress is comparable to that found in a cross-sectional study on ward nurses in a Malaysian public hospital, which reported that 25% of nurses reported a high level of perceived stress (5). The primary outcome of this study was a statistically significant reduction in stress perception scores upon completion of the mindfulness-based intervention programme. Mindfulness-based interventions provide benefits by increasing the capacity of participants to be mindful of present-moment experiences and enabling them to be consciously aware of stressful adverse events (24). Mindful attention can promote more adaptation for stress feeling and cognitive change by ‘turning down’ negative appraisal of adverse events (24). In this way, it can enable individuals to interpret circumstances as less distressing, thereby reducing perceived stress (10, 24–26). The results of the present study are consistent with those of Gauthier et al. (17), who applied a shortened version of a mindfulness-based intervention to paediatric nurses. Gilmartin et al. (8) also reported a significant reduction in stress levels after a brief mindfulness-based intervention involving healthcare providers. Hee et al. (15) reported a significant decrease in stress perception among hospital staff nurses, with a moderate effect size after a 5-week of mindfulness-based intervention programme. As reported previously, brief mindfulness-based training provides a practical self-help tool to help busy nurses manage stress in a high-stress work environment (17, 19, 26, 27). In the present study, a 1-day brief mindfulness-based intervention, together with 1 h monthly practice sessions, reduced perceived stress among nurses in HUSM. This study suggests that an intervention of short duration, combined with monthly practical session and daily online reminder may be sufficient to induce a significant decrease in perceived stress among nurses.

Post-intervention, the anxiety scores of the participants in the present study were significantly reduced. As reported previously, mindfulness skills may help prevent negative thoughts and emotions and, in turn, lead to a decrease in anxiety symptoms (28). In a previous study, mindfulness techniques known as ‘mindful body stretching’ and ‘mindful breathing’ promoted relaxation and significantly reduced symptoms of anxiety among nurses (8). Sood et al. (29) found a similar significant reduction in anxiety scores of healthcare workers after a mindfulness-based intervention. Ghawadra et al. (4, 19) reported similar findings in their studies on hospital staff nurses who took part in a 2-h MINDFULGym mindfulness-based intervention workshop, followed by 4 weeks of self-practice at home. According to Ghawadra et al. (19), by helping nurses to manage their stress and anxiety, the mindfulness practice directly improved their job satisfaction.

In contrast to the stress and anxiety scores, there was no statistically significant reduction in the depression scores in the present study. The result is consistent with that of a study by Foureur et al. (30) who reported no significant improvement in depression after a 1-day mindfulness-based intervention. These findings are not surprising, as the primary aims of mindfulness-based interventions are stress reduction and enhancement of well-being. Mindfulness-based cognitive therapy (MBCT) is mindfulness-based psychotherapy specifically developed to treat depression (30, 31). In MBCT, participants learn skills that allow them to disengage from automatic dysfunctional cognitive thinking or depression-related ruminative thinking based on a combination of mindfulness practice and cognitive behavioural theory. The intervention in the present study did not incorporate cognitive and behaviour theory (31).

According to previous research (8), after a mindfulness-based intervention, staff nurses were more attentive to the present-moment while at work, thereby preventing possible errors that could arise as a result of ‘running on automatic pilot’. Research also reported that mindfulness practice can increase awareness among nurses of the importance of self-kindness, as well as enable them to recognise difficult emotions and sensations but not react to them in a high-stress working environment (13). With such awareness, they are better able to face challenging and difficult life events and experiences, as they maintain a non-judgemental state of complete awareness of thoughts, emotions or experiences. In this way, negative cognition and self-judgment were reduced, resulting in lower scores for perceived stress.

The present study has some limitations. The most important limitation is the lack of randomisation and a control group. This reduces the level of evidence of this study. Another limitation is the use of self-report scales for data collection, as these can result in response bias. In addition, we did not measure compliance with mindfulness practice at home. As noted previously (8, 17, 18), the level of adherence to a mindfulness-based intervention has implications for its effectiveness. Finally, the sampling method used in this study was non-probability sampling. The latter relies on the researcher’s judgement in recruiting participants. This reduces the generalisability of the results.

Future research should measure the impact of the duration of mindfulness-based intervention home practice on stress levels among staff nurses, as well as on other psychological domains (e.g. burnout and job stratification). Such research should use clinician-rated questionnaires to evaluate stress, anxiety and depression.

## Conclusion

The findings of this study suggest that a brief mindfulness-based intervention for hospital staff nurses can yield meaningful outcomes, especially in terms of preventing stress and enhancing well-being among healthcare workers. The brief mindfulness-based intervention described in the present study can be easily applied in hospital setting. The findings of this study can aid hospital management wishing to put in place interventions aimed at combating work-related stress among nurses.

## Figures and Tables

**Table 1:** Sociodemographic characteristic of participants

Variable	Number (*n*) Frequency (%)	Mean (SD)
Age (years old)		34.63 (6.22)
Working experience (years)		11.09 (6.52)
Gender		
Male	2 (5.7)	
Female	33 (94.3)	
Race		
Malay	34 (97.1)	
Chinese	1 (2.9)	
Marital status		
Married	31 (88.6)	
Single/Divorce	4 (11.5)	
Number of children		
0–2	22 (62.8)	
3–4	3 (8.6)	
> 4	10 (28.6)	
Education		
Diploma	27 (77.1)	
Degree	8 (22.9)	
Post basic training		
Yes	22 (62.9)	
No	13 (37.1)	

**Table 2:** Pre- and post-intervention comparison

Variables	Pre-intervention mean (SD)	Post-intervention mean (SD)	Mean difference (95% CI)	*t*	Significant (*P*)
Perceived stress (PSS 10)	15.66 (5.75)	14.17 (4.32)	1.49 (0.06, 2.92)	2.11	0.04
Anxiety (DASS)	7.37 (6.07)	6.17 (4.40)	1.20 (0.06, 2.34)	2.14	0.04
Depression (DASS)	4.00 (6.00)#	4.00 (4.00)#	–	–	0.62*

Notes:

* Wilcoxon signed-rank test;

# median (IQR)
